# Fifteen Years of Advancing Cardiovascular Rehabilitation in Low-Resource Settings through the International Council of Cardiovascular Prevention and Rehabilitation (ICCPR) and a Look Ahead

**DOI:** 10.5334/gh.1484

**Published:** 2025-10-09

**Authors:** Abraham Samuel Babu, Sherry L. Grace, Dion Candelaria, Robyn Gallagher, Aashish Contractor, Carley O’Neill, John Buckley, Gabriela Lima de Melo Ghisi

**Affiliations:** 1 Department of Physiotherapy, Manipal College of Health Professions, Manipal Academy of Higher Education, Manipal, India; 2 KITE Research Institute, Toronto Rehabilitation Institute, University Health Network, Toronto, Canada; 3 Temerty Faculty of Medicine, University of Toronto, Toronto, Canada; 4 Faculty of Health, York University, Toronto, Canada; 5 The University of Sydney, Faculty of Medicine and Health, Susan Wakil School of Nursing and Midwifery, Sydney, Australia; 6 Sir H.N. Reliance Foundation Hospital, Mumbai, India; 7 School of Kinesiology, Faculty of Professional Studies, Acadia University, Nova Scotia, Canada; 8 School of Allied Health Professions, Keele University, Stafford, United Kingdom

**Keywords:** Cardiac Rehabilitation, low-and middle-income countries, Secondary Prevention, global health

## Abstract

Cardiovascular disease (CVD) remains the leading cause of morbidity and mortality worldwide, with a particular burden in middle-income countries (MICs). Cardiac rehabilitation (CR) is a secondary prevention model resulting in reduced CV mortality, morbidity, cost-effectively. However, CR is under-utilized globally, especially in MICs due to structural, social, and economic barriers. The International Council of Cardiovascular Prevention and Rehabilitation (ICCPR) is a World Heart Federation-affiliated umbrella association founded ~15 years ago, now comprised of 50 Associations and 30 champions in countries without CR societies. ICCPR addresses delivery challenges through: CR guidelines tailored for MICs, the Global CR Audit to support advocacy, the International CR Registry (ICRR), Program Certification to support service quality, multi-disciplinary provider training (CR Foundations Certification; CRFC), women-focused CR initiatives, and partnerships with the World Health Organization. ICCPR continues to foster global CR accessibility through collaboration, communication, as well as research and advocacy with their upcoming Global CR Audit Update.

## Introduction

Despite significant advances in medical science, cardiovascular disease (CVD) remains the leading cause of morbidity and mortality worldwide (1). Cardiac rehabilitation (CR) is an established multi-component model of secondary prevention that targets all CV risk factors–including exercise training, patient education, and psychosocial counselling–to reduce the sequelae of CVD and optimize patient function (2). Cochrane reviews demonstrates that participation in CR significantly reduces cardiovascular mortality by 26%, hospitalizations by 23%, and myocardial infarction by 18% in individuals with coronary heart disease (3). Similar benefits have been observed among individuals with heart failure, among other cardiac conditions (4).

Despite these well-established benefits, CR remains greatly underutilised and insufficiently implemented around the globe (5). This gap is most pronounced in middle-income countries (MICs), where 14.8 million more CR “spots” are needed annually to treat incident ischemic heart disease patients alone (5,6). Nevertheless, CR access remains grossly insufficient and is highly inequitable in high-income countries, hence, the focus should be on ‘low-CR resourced’ settings (7).

Therefore, it is crucial to address the persistent gaps in CR accessibility and delivery, particularly in low-resource settings where structural, socioeconomic and other barriers hinder implementation (7). The International Council of Cardiovascular Prevention and Rehabilitation (ICCPR) was conceived in 2010 to address these gaps. In this paper, we summarize the past efforts and future goals of the ICCPR community to meet this great need.

## International Council of Cardiovascular Prevention and Rehabilitation (ICCPR)

In 2010, groundwork began to establish ICCPR, laying the foundation for its mission to unite national/regional CR, clinical Associations, Foundations or other CR-related organizations worldwide to promulgate efforts in promoting CR. In 2013, the ICCPR was formally launched following approval by the World Heart Federation (WHF) and the publication of its charter (8), marking a significant milestone in promoting and advancing global CR efforts.

**Figure 1:**
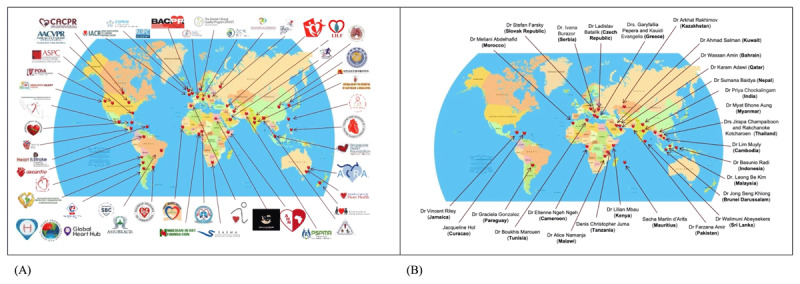
Global distribution of ICCPR member organiations **(A)** and “champions” **(B)**.

ICCPR is a not-for-profit organization, which is formally a member of the World Heart Federation. Today, ICCPR has 50 member organizations and 30 “Champions” (in countries without formal CR Societies or related Associations who have named a Board Member to our Council; Figure 1), such that its reach globally spans 161 countries, including 90 low- and middle-income countries, from all 6 World Health Organization (WHO) regions. Indeed, numerous champions are currently working with the support of ICCPR to formalize an inaugural CR working group in their jurisdiction. By leveraging synergies, ICCPR seeks to foster CR awareness, capacity-building, to promulgate implementation of national/regional initiatives in other jurisdictions, generate needed knowledge and support policy development to enhance quality CR implementation internationally.

## ICCPR’s Fifteen-Year Journey

**Figure 2:**
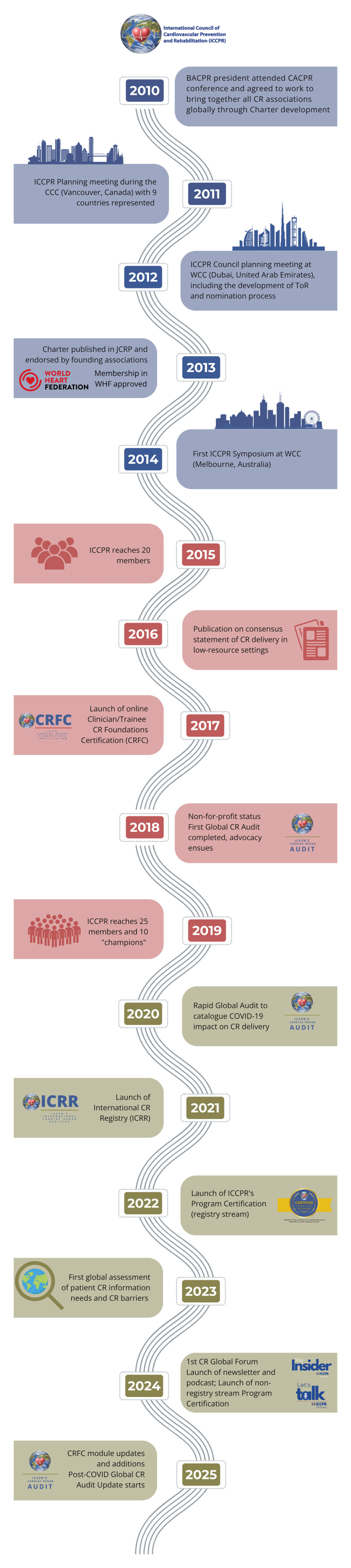
Timeline highlighting main ICCPR activities in the past fifteen years. BACPR, British Association of Cardiovascular Prevention and Rehabilitation; CACPR, Canadian Association of Cardiovascular Prevention and Rehabilitation; CCC, Canadian Cardiology Conference; COVID-19, COronaVIrus Disease of 2019; CR, cardiac rehabilitation; CRFC, cardiac rehab foundation certification; ICCPR, International Council of Cardiovascular Prevention and Rehabilitation; JCRP, Journal of Cardiopulmonary Prevention and Rehabilitation; WCC, World Cardiology Conference; WHF, World Heart Federation.

The following timeline highlights the activities of ICCPR and summarises its impact over the last fifteen years (Figure 2).

### 2010–2014: Establishing ICCPR and Uniting Global CR Efforts

The journey to establish ICCPR began in 2010, driven by a shared vision to unite national and regional CR associations worldwide. From 2010 to 2014, foundational milestones included a scoping review to identify international CR organizations, developing the ICCPR charter, and engaging stakeholders through meetings and presentations at major conferences, including those from foundation members (AACVPR, BACPR and CACPR). A council planning meeting at the 2012 World Cardiology Conference in Dubai (United Arab Emirates) finalized governance structures and ICCPR’s application for membership with the WHF. By 2013, the charter was published (8), WHF membership approved, and ICCPR’s website launched. The period culminated in 2014 with the first ICCPR symposium at the WCC in Melbourne (Australia), solidifying its global leadership.

### 2014–2019: Advancing Global CR through Consensus, Advocacy, and Capacity Building

At the time of ICCPR’s formation, existing CR guidelines were primarily developed by organisations from high-income countries (9), with only the 1993 WHO report considering the unique challenges faced by low-resource settings (10). Based on recommendation of our members, ICCPR’s first major initiative was to develop a consensus statement on CR tailored for low-resource settings (11,12). The comprehensive model of care incorporated key internationally-agreed components (9) such as initial assessment, lifestyle risk factor management, medical risk factor management, patient education, psychosocial counselling, and outcome evaluation. Since publication, there have been numerous randomized trials in the field of CR in MICs (13), such that this consensus statement could now be updated as a clinical guideline.

Also during this period, members expressed a need for advocacy tools to advance CR in their jurisdictions (14). In response, ICCPR investigated CR reimbursement models associated with greater delivery capacity and identified key strategies to support CR advocates in achieving concordant policy changes. ICCPR launched an advocacy toolkit to empower stakeholders with templates and strategies to lobby for CR-supportive policy and funding (https://globalcardiacrehab.com/Advocacy).

Again, based on member assertions regarding the need to build health human resource capacity to deliver CR (5,15), ICCPR launched the Cardiac Rehabilitation Foundations Course (CRFC)–an 8-hour online training program featuring a module for each core CR component led by experts from across the globe. The CRFC has been shown to equip multidisciplinary clinicians and trainees alike with the knowledge essential for effective comprehensive CR delivery, which they apply in practice (16). To January 2025, there have been 2,385 learners from 46 countries. Most recently, national and regional CR associations have embarked on initiatives to train one staff member per program in their respective jurisdictions, though group pricing (e.g., Australia, Africa, Pakistan, India). The latest recurring peer review resulted in updates to several modules as well as addition of optional sub-modules on sexuality and access/utilization.

### 2020–Present: Advancing CR Globally Through Measurement and Quality Improvement

The International CR Registry (ICRR) was also launched by ICCPR in response to member request for a mechanism to systematically assess their program outcomes and benchmark their quality relative to other programs in low-resource settings worldwide (17). It is the first international CR registry (18), tailored based on the needs of programs in low-resource setting (19). Since its inception in late 2021 during the latter stages of the COVID-19 pandemic, the ICRR has expanded significantly, now comprising ~25 sites across every WHO region, with over 6,000 participants enrolled. This, coupled with quality improvement initiatives by the ICRR sub-committee, has supported participating sites to identify and implement strategies to enhance the effectiveness and efficiency of their CR programs (20).

In addition, ICRR-participating programs have the opportunity to be considered for certification by ICCPR (21), recognizing them for meeting an internationally-agreed standard with regard to program structure, processes and outcomes. Indeed, the certified program in Qatar received recognition of their achievement from not only the top administration of their institution, but also their Minister of Health who then approved their proposal for satellite expansion. Most recently, ICCPR launched a non-registry stream of program certification to expand access to programs interested in earning this recognition (https://globalcardiacrehab.com/Program-Certification-Apply).

ICCPR also conducted the first Global CR Audit (2016/2017) and a rapid re-assessment at the outset of the COVID pandemic. The 2016/17 Global CR Audit engaged over 1,000 CR programs across 93 countries (5), providing a first-of-its-kind comparison of CR capacity and disease incidence. This audit highlighted the global disparities in CR availability and informed ICCPR’s subsequent initiatives. A Rapid Audit was conducted in spring 2020 during the COVID-19 pandemic (22), revealing that three-quarters of CR programs had ceased operations or shifted to low-tech, unsupervised models. Building on these insights, the ICCPR community identified the need for an updated audit to reflect current challenges and advances in CR services. This update, launching in 2025, aims to provide updated data and further inform global CR advocacy and capacity-building efforts.

Recognizing the unique needs of certain under-served populations in CR, ICCPR has taken a leading role in advocating and promoting the need for women-focused CR initiatives. This has included development of the first-ever Women-focused Clinical Guidelines for CR, global surveys on information needs and CR barrier assessment, and strategies to expand women-focused CR delivery (23,24,25).

Furthermore, ICCPR has been instrumental in supporting the WHO development of the Package of Rehabilitation Interventions ischemic heart disease (26). This package provides a comprehensive framework for addressing the rehabilitation needs of patients, focusing on essential components such as lifestyle modifications, exercise, and psychosocial support. ICCPR’s partnership with WHO also extends to the technical leadership of the WHO Package, with Prof Grace leading the working group in 2018.

Another key achievement of ICCPR is its pivotal role in advancing global collaboration in CR. Together with SOLVE-CHD (https://solvechd.org.au/), ICCPR co-organized the inaugural Global Forum on Cardiac Prevention and Rehabilitation, held on August 2024, in London, United Kingdom. This historic event brought together 52 global experts and emerging leaders from over 40 organizations and 20 countries, with representation from all WHO regions. Insights from a pre-forum survey, with 327 responses globally, guided the forum’s agenda, emphasizing capacity building, addressing care gaps, and enhancing awareness among policymakers and providers. The outcomes of this forum, to be published in a peer-reviewed journal, highlight ICCPR’s enduring commitment to uniting stakeholders, strengthening global CR networks, and driving equitable access and quality improvement across all regions.

ICCPR has significantly expanded its communication and outreach efforts to connect with the global CR community. Through its program email distribution list, which includes over 2,000 contacts worldwide, ICCPR ensures that crucial updates, resources, and advocacy tools reach stakeholders in all regions. Complementing this, ICCPR’s social media platforms boast a reach of over 10,000 followers, fostering engagement and promoting CR awareness on a global scale. In October 2024, ICCPR launched its monthly newsletter to provide a regular and concise overview of key developments, best practices, and opportunities in CR. Furthering this mission, ICCPR introduced a podcast in November 2024, offering an accessible platform for in-depth discussions on emerging CR topics, innovations, and personal stories. Together, these initiatives enhance knowledge sharing, build stronger global networks, and empower healthcare providers, advocates, and policymakers to advance CR delivery and equity worldwide.

## Conclusion

ICCPR aims to support the growth and enhancement of CR globally, particularly in low-resource settings. In staying true to its’ mission, the ICCPR continues to bring together the global CR community to share resources, learnings and promulgate efforts. They continue to build the capacity of healthcare providers in CR through online certification and improve the quality of CR delivered by encouraging participation in the ICRR and their program certification scheme. Furthermore, ICCPR looks forward to detailing CR availability, capacity, density and unmet need in every region globally. This will be achieved by our strong network of Global CR Audit Update champions, who will be supported to leverage findings for local advocacy efforts. We remain steadfast in our commitment to collaborating with those dedicated to advancing the CR mission. By uniting our efforts, we can accelerate progress toward achieving universal access to high-quality CR services for all individuals in need, ensuring a healthier future for communities worldwide.
